# Safety and Tolerability of a Shorter Agalsidase Beta Infusion Time in Patients with Classic or Later-Onset Fabry Disease

**DOI:** 10.3390/biomedicines12112578

**Published:** 2024-11-11

**Authors:** Dominique P. Germain, Alice Porto Vasconcelos, Lien Tran Thi Phuong, Najya Bedreddine, Mihaela Turcan, Wenting Trang, Lynda Barache

**Affiliations:** 1 Division of Medical Genetics, University of Versailles-St Quentin en Yvelines (UVSQ), Paris–Saclay University, 2 Avenue de la Source de la Bièvre, F-78180 Montigny, France; 2 Referral Center for Fabry Disease and Lysosomal Diseases, MetabERN, F-92380 Garches, France; 3 Division of Medical Genetics, APHP Paris Saclay University, F-92380 Garches, France; 4 Association des Patients de la Maladie de Fabry, F-21160 Perrigny les Dijon, France

**Keywords:** Fabry disease, agalsidase beta, quality of life, reduced infusion time, safety, tolerability

## Abstract

Background: The multisystem manifestations of Fabry disease can create major challenges in patient care. Although enzyme replacement therapy with recombinant agalsidase beta has demonstrated clinical benefits, the standard fortnightly, multi-hour infusion regimen imposes a substantial burden on patients. Methods: We assessed the safety and feasibility of shortening the agalsidase beta infusion time to 90 min in adult patients with classic or later-onset Fabry disease in the absence of premedication. A total of 39 consecutive adult patients (agalsidase-naïve: n = 7; with significant comorbidities: n = 15) with no recent infusion-associated reactions underwent a total of 85 agalsidase beta infusions in our tertiary reference centre for lysosomal diseases. Each infusion was administered at a constant rate (between 0.78 and 1.17 mg/min, depending on the total dose administered). Results: No adverse events of any type (including discomfort and infusion-associated reactions) were reported during or after infusions. The patients’ vital signs and physical examination remained stable, and patients’ satisfaction was high. Conclusions: Our results suggest that shortening the agalsidase beta infusion time to 90 min is safe and feasible in stably treated adult patients with Fabry disease and no recent infusion-associated reactions.

## 1. Introduction

Fabry disease (FD, OMIM #301500) is an X-linked genetic metabolic disease caused by pathogenic variants in the *GLA* gene (HUGO Gene Nomenclature Committee ID: 4296; Gene Entrez: 2717; NCBI reference sequence: NM_000169.3) and thus a deficiency in activity of the lysosomal alpha-galactosidase A (α-Gal A, Enzyme Commission number: EC 3.2.1.22; UniProt ID: P06280). This deficiency results in the progressive accumulation of globotriaosylceramide (Gb_3_) and its deacylated derivative globotriaosylsphingosine (lyso-Gb_3_) in the lysosomes of various tissues and organs, leading to multisystem manifestations, such as kidney failure, hypertrophic arrhythmogenic cardiomyopathy, and cerebrovascular events [1,2,3,4]. Starting in 2001, enzyme replacement therapy (ERT) [5,6] has significantly improved the care and lives of people with FD by reducing substrate accumulation and thus slowing disease progression. A pharmacological chaperone, migalastat, has also been approved since 2016 by the Food and Drug Administration (FDA) and the European Medicines Agency (EMA) as an oral therapy, with its use restricted to patients bearing amenable pathogenic variants of *GLA* [7].

Although gene-activated agalsidase alfa (Replagal^®^, Takeda Pharmaceutical, Tokyo, Japan) is administered fortnightly by intravenous infusion over 40–60 min [1,6,8,9,10,11,12,13] at a rate between 0.23 mg/min (if infused in 60 min) and 0.35 mg/min (if infused in 40 min), recombinant agalsidase beta (Fabrazyme^®^, Sanofi, Framingham, MA, USA) is typically administered intravenously (again fortnightly) over a longer period [5,10,14,15,16,17,18,19,20], typically within 2 h 30 min (0.47 mg/min) but often up to 3.5 h (0.33 mg/min) in clinical practice. The more recently EMA- and FDA-approved pegunigalsidase alfa (Elfabrio^®^, Chiesi Pharmaceutici, Parma, Italy) [21,22,23,24,25], which could be infused in 60 min (1.17 mg/min) during the extension study of the BALANCE clinical trial upon decision of the investigator, should be administered during a minimal time of 90 min (0.78 mg/min), and only after four mandatory infusions in a hospital setting, per FDA decision.

The European Medicines Agency’s summary of product characteristics (SPC) for agalsidase beta (Fabrazyme^®^) states that the infusion rate should be no more than 0.25 mg/min (15 mg/h) initially to minimize the potential for infusion-associated reactions (IARs), but can be increased once patient tolerance has been established [26]. Hence, depending on the patient’s bodyweight, the therapeutic dose of ERT may need to be delivered over a long period. However, in the absence of IARs or other adverse events, a lengthy infusion regime poses a significant burden on patients’ quality of life and might reduce treatment adherence and therefore effectiveness. Hence, shortening and optimizing infusion protocols to reduce treatment burden while maintaining safety and effectiveness is an area of ongoing research and clinical interest.

A few research groups have described their attempts to reduce the agalsidase beta infusion time safely and effectively. In 2007, a phase IV double-blind, randomized placebo-controlled trial reported on decreasing the infusion time to 90 min in 51 patients treated with agalsidase beta or placebo. However, all the participants received pretreatment with paracetamol or ibuprofen, and some were given an antihistamine. Twenty-eight of the patients reported mild or moderate IARs [27].

The use of a shortened agalsidase beta infusion protocol was reported in a Spanish series of six cases (five women and a man) [28]. The first four to eight infusions were administered over several hours (at an initial, fixed rate of 0.25 mg/min) but subsequent infusions were gradually shortened to as little as 45 min in a hospital setting. Premedication with 1 g paracetamol was provided, and patients were closely monitored during the infusion. The outcomes were favourable, and only one patient (a woman with a history of allergies) experienced an IAR after the tenth (45-min) infusion. The IAR was treated with antihistamines and corticosteroids [28].

An Italian study reported retrospectively on a stepwise infusion rate escalation protocol in a cohort of 53 patients with FD [29]. The initial, standard infusion duration was as long as 7 h in the heaviest patients. Patients experiencing an IAR were premedicated before subsequent infusions. Fifty-two of the 53 patients were able to move to a shorter infusion, and this duration was below 2 h in 38 patients. The mean ± standard deviation (SD) infusion duration was significantly shorter in previously treated patients than in ERT-naïve patients (100.91 ± 15.14 min vs. 130.71 ± 38.53 min, respectively; *p* < 0.01) [29].

In 2022, the start of a multicentre Italian prospective study with a dose infusion rate of 15 mg/h for the first four months was described [30]. Patients experiencing an IAR were premedicated before subsequent infusions. During the following four infusions, the infusion rate was increased progressively from 15 to 35 mg/h, giving the shortest infusion time at the end of the sixth month [30]. The study’s results were published in 2024: 25 of the 31 enrolled patients were treatment-naïve, and the other 6 had been switched from agalsidase alfa to agalsidase beta. Only 1 patient experienced a (mild) IAR, and 28 of the patients were seronegative at month 12 [31].

In a report published in 2023, a Japanese post-marketing study described the impact of short (<90 min) infusions versus standard (≥90 min) infusions on various safety variables in children and adults with FD [32]. The presence or absence of premedication was not reported. The incidence of serious adverse events was low (less than 1%) in patients weighing less than 30 kg, and the frequency of such events in given individuals fell over time. The authors concluded that gradual reductions in infusion times may be feasible in patients who tolerate treatment well [32].

Despite these valuable contributions, critical questions persist with regard to the initiation of shorter infusion times. To address these knowledge gaps, we evaluated the tolerability of a shortened infusion time for agalsidase beta in adult ERT-naïve and ERT-experienced patients with classic or later-onset FD. The safety of administering agalsidase beta over a 90-min infusion for all FD patients in the absence of recent infusion associated reaction was the primary objective. Specifically, we assessed clinical variables and patient-reported treatment satisfaction and investigated the feasibility of reducing the infusion duration from 2.0–3.5 h to 90 min in routine clinical care under strict medical supervision in our tertiary referral centre, without premedication in adult patients at various stages of disease progression and severity, in the absence of recent infusion-associated reactions.

## 2. Patients and Methods

Between March 2023 and May 2024, 39 consecutive adult patients with genetically confirmed FD followed at our European Reference Network (ERN) tertiary centre for lysosomal diseases (www.centre-geneo.com) were offered to benefit from a reduced agalsidase beta infusion time in a routine care setting (90 min as per the summary of product characteristics). Most patients had been treated at-home with historically fortnightly 3.5-h gradually decreased to 2.0-h-long intravenous infusions of agalsidase beta, with the exception of seven patients who were naïve to enzyme replacement therapy. Any ongoing or recent agalsidase beta infusion-associated reaction was an exclusion criterion, as was a medical history of IgE antibodies to agalsidase beta at any time in the patient medical history [33]. Patients under 16 years of age and pregnant or lactating women were not considered for participation.

Baseline characteristics (including age, sex, *GLA* variant, bodyweight, and medical history of comorbidities) were recorded for each patient (Table 1). For all patients, the infusion duration was set to 90 min (as mentioned in the SPC of agalsidase beta) without the administration of premedication. This was performed in the setting of our tertiary referral centre and, for the seven patients naïve to ERT, under the strict medical supervision of a medical doctor present at the patient’s bedside during the 90 min. Once an infusion had started, the infusion rate was not modified. Vital signs (including body temperature, blood pressure, and resting heart rate) were measured before and after the infusion. Any IARs or other adverse events reported by the patient or the attending healthcare professionals were documented (Table 2).

Descriptive statistics were used to summarize patient demographics, genotype characteristics, infusion parameters, and safety outcomes. Continuous variables were reported as the mean ± SD, mean (range), or the median [interquartile range (IQR)], as appropriate. Categorical variables were expressed as the frequency (percentage). Given the descriptive nature of this study, no formal hypotheses were tested.

## 3. Results

Thirty-nine consecutive adult patients (21 males and 18 females) bearing 29 different pathogenic *GLA* variants (Table 2) were scheduled for a hospital visit in routine clinical care. The mean (range) age was 50.4 (24–78), and the mean bodyweight was 73.3 kg. Seven of the 39 patients were ERT-naïve and benefited from constant medical surveillance at their bedside during their infusions. Fifteen patients presented with significant comorbidities: these included end-stage renal disease with the requirement for dialysis or kidney transplantation, a history of stroke and/or transient ischaemic attack, hypertrophic cardiomyopathy, atrial fibrillation, an elevated body weight requiring 105 mg of agalsidase beta (n = 5) or a history of IARs (though not ongoing) (Table 2).

For a total of 85 infusions, the mean dose of agalsidase beta infusion was 70.8 mg, delivered over a mean period of 87.2 min; this yielded a mean infusion rate of 0.83 mg/min. The highest agalsidase beta infusion rate recorded in the setting of our routine regimen was 1.17 mg/min, which corresponds to 105 mg of agalsidase beta infused over 90 min in patients with higher body weight (>95 kg (Table 1). Anecdotally, the ultimate highest agalsidase beta infusion rate was 1.40 mg/min in the three patients who, for various imperative reasons or personal convenience, requested a shorter infusion time under strict reinforced medical supervision (70 mg of agalsidase beta infused over 50 min), without any side effect (Table 2).

All 39 participants tolerated the shorter infusions well, with no reports of adverse events (not even discomfort or IARs) during or following any of the procedures. The vital signs remained stable throughout the infusion process, with no clinically significant changes. The level of patient satisfaction was high (Table 3).

## 4. Discussion

Our present data on clinical variables and vital signs showed that it was feasible to reduce the agalsidase beta infusion duration to 90 min (as outlined in the summary of product characteristics of agalsidase beta) in routine clinical care, without the need for premedication and based on individual patient requirements. Anecdotally, an infusion time below 90 min was specifically requested by patients in a few imperative cases and then applied under strict medical supervision. Importantly, no adverse events or IARs were reported during or after for the 85 infusions administered, and the patients’ vital signs remained stable.

Although our single-centre case series differed from previous studies in this field in several respects, we confirmed the overall literature findings on the safety and tolerability of shorter agalsidase beta infusions. While we assessed a specific 90-min infusion protocol in most patients, Spanish authors tried to shorten the infusion time even more—to as little as 45 min [28]. Similarly, in the small subset of patients (n = 7) who, for personal convenience or imperative reasons on the day of the infusion, requested it from us, further shortening of the infusion time to either 60 min (n = 4) or 50 min (n = 3) under strict medical supervision (medical doctor present at patient’s bedside) was performed without any adverse event. Although some of the patients studied by Italian colleagues were able to move to infusions lasting between 90 and 120 min, the study’s main focus was the efficacy and safety of a fortnightly infusion regimen of initially up to 7 h (for the heaviest patients) [29] that reflected the standard practice from the time of the clinical development program of agalsidase beta [5]. In contrast, our study sought to explore the feasibility and safety profile of shorter infusion durations, specifically targeting a 90-min infusion regimen. Our present results are also partially or fully in line with several findings of a previous Japanese study [32]. The authors observed a non-significant trend towards fewer adverse events in patients weighing less than 30 kg. Likewise, we did not observe obvious disparities in safety outcomes as a function of the patient’s bodyweight or comorbidities. It is noteworthy that the few published studies on this topic were conducted in distinct populations and settings but provide complementary insights into the safety and feasibility of shorter agalsidase beta infusions durations in FD patients receiving enzyme therapy [31]. Our present findings contribute to the growing body of evidence in favour of moving towards the patient-centred treatment of rare lysosomal disorders, including home-based treatment.

Our study had several strengths. Firstly, we included consecutive (but recent IAR-free) patients, which reduced selection bias. Some of the study participants (n = 7) were agalsidase-naïve, others suffered from advanced FD and/or major comorbidities (such as heart failure and kidney failure), and yet others had a past history of allergic reactions; this heterogeneity illustrated the potential for shortening the infusion time in a real-life patient population. Secondly, premedication was not administered.

Our study also had several limitations. Firstly, the sample size (n = 39 patients) was relatively small. Secondly, the study duration was short; hence, further investigation will be needed to validate the long-term safety of optimized infusion protocols. Thirdly, we did not use a validated questionnaire to assess the patients’ level of satisfaction with the shortened infusion. Interestingly, studies conducted in vitro allow for the identification of relatively stable alpha-galactosidase variants that could be present in patients. Several papers report residual activity of missense variants, which could be useful for predicting outcomes [34,35,36,37]. In the future, longer-term multicentre studies should address these points and explore safety, immunological variables [16], and patient-centred outcomes (including quality of life) in individuals receiving optimized agalsidase beta infusion protocols that, for safety reasons, have been implemented only in our tertiary referral centre for now (http://www.centre-geneo.com) and not yet transitioned to a home care setting.

In conclusion, our results support the safety and feasibility of shortening the agalsidase beta infusion time to 90 min in non-premedicated IAR-free, adult patients at various stages of FD. It should be emphasized that our study population may have been less likely to develop IARs since after eight to twelve administrations of ERT, the likelihood of IARs decreases. However, after more than twenty years of routine administration of agalsidase beta, our study aimed at contributing to optimize regimen, increase flexibility, reduce the treatment burden, improve patient adherence, and ultimately improve the patient-centred management of this complex metabolic disorder [38].

## Figures and Tables

**Table 1:** Patients’ demographics.

Patients			
**Sex**	Female, N = 18 *^1^*	Male, N = 21 *^1^*	Overall, N = 39 *^1^*
**Age *^1^***	54.83 (13.81) [32, 78]	46.52 (11.88) [24, 73]	50.36 (13.31) [24, 78]
**Weight *^1^***	66.60 (11.76) [48, 95]	78.90 (12.74) [57, 106]	73.23 (13.64) [48, 106]
***GLA*** **genetic variants**			
**Deletion**	0/18 (0%)	1/21 (4.8%)	1/39 (2.6%)
**Frameshift**	0/18 (0%)	4/21 (19%)	4/39 (10%)
**Indel**	0/18 (0%)	1/21 (4.8%)	1/39 (2.6%)
**Missense**	15/18 (83%)	13/21 (62%)	28/39 (72%)
**Nonsense**	3/18 (17%)	1/21 (4.8%)	4/39 (10%)
**Splicing**	0/18 (0%)	1/21 (4.8%)	1/39 (2.6%)
**Phenotype**			
**Classic**	17/18 (94%)	20/21 (95%)	37/39 (95%)
**Late-onset**	1/18 (5.6%)	1/21 (4.8%)	2/39 (5.1%)
**Significant comorbidities**	6/18 (33%)	11/21 (52%)	17/39 (44%)
**Naïve**	3/18 (17%)	3/21 (14%)	6/39 (15%)
**Infusions**			
**Sex**	Female, N = 38 *^1^*	Male, N = 47 *^1^*	Overall, N = 85 *^1^*
**Time (min)**	86.32 (11.01) [50, 90]	87.87 (8.32) [50, 90]	87.18 (9.59) [50, 90]
**50**	2/38 (5.3%)	1/47 (2.1%)	3/85 (3.5%)
**60**	2/38 (5.3%)	2/47 (4.3%)	4/85 (4.7%)
**90**	34/38 (89%)	44/47 (94%)	78/85 (92%)
**Dose (mg)**	66.32 (10.89) [35, 70]	74.47 (11.81) [70, 105]	70.82 (12.05) [35, 105]
**35**	4/38 (11%)	0/47 (0%)	4/85 (4.7%)
**70**	34/38 (89%)	41/47 (87%)	75/85 (88%)
**105**	0/38 (0%)	6/47 (13%)	6/85 (7.1%)
**Rate (mg/min)**	0.79 (0.21) [0, 1]	0.86 (0.17) [1, 1]	0.83 (0.19) [0, 1]
**0.39**	4/38 (11%)	0/47 (0%)	4/85 (4.7%)
**0.78**	30/38 (79%)	37/47 (79%)	67/85 (79%)
**1**	0/38 (0%)	1/47 (2.1%)	1/85 (1.2%)
**1.17**	2/38 (5.3%)	8/47 (17%)	10/85 (12%)
**1.4**	2/38 (5.3%)	1/47 (2.1%)	3/85 (3.5%)

*^1^* Mean (SD) [Range]; n/N (%).

**Table 2:** Summary of vital signs of patients before and after 90-min-infusions of agalsidase beta.

Patient ID	Sex	Phenotype			Weight (kg)	Dose (mg)	Time (min)	Rate (mg/ min)	Vitals Before Infusion			Vitals After Infusion			History of IAR	
			Pathogenic Variant	Average Time Since the Start of ERT (Years)					BT (°C)	BP (mmHg)	HR (bpm)	BT (°C)	BP (mmHg)	HR (bpm)		IAR
*P#1*	M	Classic	p.G183D	22	63.5	70	90	0.78	36.4	118/86	73	36.5	117/88	72	No	No
					59			0.78	36.8	131/82	75	36.1	124/81	77		No
					58			0.78	36.9	131/87	74	36.8	123/70	72		No
*P#2*	F	Classic	p.C52C	8	67.3	70	90	0.78	36.1	106/74	76	36.2	110/75	74	No	No
*P#3*	F	Classic	p.W236R	2	66	70	90	0.78	36.4	106/75	62	36.5	104/75	63	No	No
*P#4*	F	Classic	p.I91T	0	90	70	90	0.78	36	126/79	78	36.2	111/79	75	No	No
					90	70	90	0.78	36.1	121/68	76	36	134/81	81		No
					90	70	90	0.78	36	132/89	66	36.8	110/76	75		No
					90	70	90	0.78	36.3	121/85	62	36.3	112/76	66		No
					90	70	90	0.78	36.6	111/70	75	36.3	127/88	82		No
					91	70	90	0.78	36.2	124/82	73	36.1	126/80	70		No
*P#5*	M	Classic	p.R227Q	7	69	70	90	0.78	36.2	113/80	62	36.1	110/87	60	No	No
*P#6*	M	Classic	p.R112C	5	80	70	90	0.78	36.5	129/93	85	36.9	145/90	70	No	No
					79	70	90	0.78	36.4	106/68	68	36.4	126/78	77		No
					79	70	90	0.78	36.4	115/80	73	36.6	125/83	66		No
					79	70	90	0.78	36.4	138/88	75	36.6	135/87	75		No
					79	70	90	0.78	36.5	118/81	67	36.5	126/80	66		No
					79	70	90	0.78	36.3	133/79	88	36.3	132/80	81		No
					78	70	70	1.00	36.6	119/84	71	36.4	121/81	71		No
*P#7*	F	Classic	p.P40L	5	53	35	90	0.39	37	110/58	58	37	95/48	59	No	No
					54	70	90	0.78	36.4	101/47	53	37.3	111/64	57		No
					54	35	90	0.39	36.8	113/68	57	36.6	98/55	61		No
					54	70	90	0.78	36.9	108/48	93	37	100/51	47		No
					54	35	90	0.39	36	97/56	68	37	101/53	57		No
					54	70	90	0.78	36.5	103/48	66	36	98/55	68		No
					55	35	90	0.39	36.6	110/51	59	37.1	94/61	52		No
					55	70	90	0.78	36.3	104/54	78	36	107/49	69		No
					55	70	50	1.40	36.6	106/70	63	36.4	112/74	65		No
*P#8*	F	Classic	p.N34H	10	95	70	90	0.78	36	157/90	71	36.3	149/96	67	No	No
					96	70	90	0.78	36.2	146/80	63	36.1	140/80	66		No
*P#9*	F	Classic	p.N34H	3	70	70	90	0.78	36.8	114/64	73	36.9	118/68	79	No	No
*P#10*	F	Classic	p.D93N	0	78	70	90	0.78	36.6	120/84	65	36.5	120/85	60	No	No
					75	70	90	0.78	36	122/63	63	36.6	127/87	57		No
*P#11*	F	Classic	p.R220*	5	57	70	90	0.78	36	118/76	62	36.3	132/60	74	No	No
					57	70	90	0.78	36	104/78	60	36.4	98/49	69		No
					58	70	90	0.78	36.5	105/58	60	36.4	121/53	60		No
*P#12*	M	Classic	p.D93N	0	80	70	90	0.78	36	142/73	61	36	142/73	61	No	No
					80.5	70	90	0.78	36	138/81	63	36.5	148/83	89		No
					80	70	90	0.78	36	130/74	57	36	139/80	59		No
					80	70	90	0.78	36.1	132/77	58	36	138/78	59		No
					80	70	90	0.78	36.2	130/77	55	36.7	132/78	54		No
					84	70	80	0.88	35	130/75	62	36.4	132/76	60		No
*P#13*	F	Classic	p.P40L	2	70	70	90	0.78	36.6	131/63	62	37.1	129/78	64	No	No
					68	70	90	0.78	36.7	125/78	63	36.4	150/64	58		No
					68	70	90	0.78	36.4	146/72	50	36.7	131/90	53		No
*P#14*	M	Classic	p.W262Lfs* 4	23	93	105	90	1.17	36.2	117/89	75	36.5	149/98	68	No	No
*P#15*	M	Classic	p.W262Lfs* 4	17	80	70	90	0.78	36	161/75	64	36.1	153/87	57	No	No
						70	90	0.78	36.3	141/84	60	36.2	135/68	65		No
*P#16*	F	Classic	p.E358G	16	55	70	90	0.78	36	135/77	55	36.8	141/72	58	No	No
*P#17*	F	Later-onset	p.N215S	5	56	70	90	0.78	36.3	104/64	66	36.6	100/64	74	No	No
					56	70	90	0.78	36.4	109/74	79	36.7	98/68	72		No
					56	70	90	0.78	37	95/66	70	36.9	88/64	65		No
					56	70	90	0.78	36.88	98/71	63	36.7	100/71	64		No
*P#18*	M	Later-onset	p.F113L	7	106	105	90	1.17	36.5	124/77	61	36.4	124/74	60	No	No
*P#19*	M	Classic	p.N264A	17	74	70	90	0.78	36	113/74	57	36.5	140/82	51	No	No
*P#20*	M	Classic			78	70	90	0.78	36	142/84	56	36.1	133/118	52	No	No
					80	70	90	0.78	36.4	149/99	54	36.4	153/93	55		No
					76	70	60	1.17	36.2	136/79	50	36.7	138/80	52		No
*P#21*	M	Classic	p.Q327K	23	93	105	90	1.17	36.5	131/76	52	36.7	137/62	53	No	No
					93	105	90	1.17	36	139/84	53	35.9	148/85	56		No
*P#22*	M	Classic	p.A156T	7	71	70	90	0.78	36.3	93/72	66	36.1	94/75	65	No	No
*P#23*	M	Classic	p.G361Rfs*	24	85	70	90	0.78	36	109/84	51	36.3	98/57	48	No	No
*P#24*	M	Classic	p.W236R	10	60	70	90	0.78	36.2	112/64	65	36.6	110/64	62	No	No
*P#25*	M	Classic	p.C382Y*15	12	70	70	90	0.78	36	121/66	83	36.7	115/55	68	No	No
*P#26*	M	Classic	p.R100T	10	57	70	90	0.78	36.4	102/66	64	36.5	105/66	68	No	No
*P#27*	F	Classic	p.F50L	10	74	70	60	1.17	36.7	124/82	57	36.3	123/81	59	No	No
						70	90	0.78	36.6	120/77	60	36.7	139/89	61		No
*P#28*	F	Classic	p.F50L	10	75	70	90	0.88	36.7	124/82	57	36.5	125/80	56	No	No
		Classic			75	70	90	0.88	37,1	110/63	62	36.7	120/65	61		No
*P#29*	F	Classic	p.C52R	16	60	70	90	0.88	35,9	107/72	78	36.3	109/73	68	Yes	No
									36.8	122/94	72	36.4	147/94	64		No
*P#30*	F	Classic	p.E358K	19	74	70	60	1.17	36.2	139/84	76	36.3	140/83	71	No	No
*P#31*	M	Classic	NA	22	94.5	105	90	1.17	36	143/93	72	36.5	144/95	70	No	No
*P#32*	F	Classic	p.G183D	17	47.5	70	90	0.78	36.5	141/80	75	36.1	122/69	60	No	No
*P#33*	M	Classic	p.E358del	0	78	70	90	0.78	36.2	114/67	55	36.1	117/71	49	No	No
						70	90	0.78	36.5	132/95	56	36.5	128/77	60		No
						70	90	0.78	36.8	142/92	58	36.2	144/85	65		No
						70	90	0.78	36.4	122/70	55	36.4	122/70	55		No
						70	90	1.00	36.4	131/83	61	36.8	132/82	71		No
*P#34*	F	Classic	p.C202*	10	57	70	50	1.40	36.5	110/69	60	36.3	119/71	62	No	No
*P#35*	M	Classic	p.D266E	5	71	70	50	1.40	36	110/89	83	36.2	109/79	84	No	No
*P#36*	F	Classic	p.C52R	17	80	70	90	0.78	36.5	121/80	78	36.6	123/81	75	No	No
*P#37*	M	Classic	p.R342*	16	70	70	60	1.17	36.2	132/82	73	36.3	131/76	68	No	No
*P#38*	F	Classic	p.Q306*	15	64	70	90	0.78	36.8	109/73	61	36.7	106/64	68	No	No
*P#39*	M	Classic	p.R112C	22	94	105	90	1.17	35.8	131/83	51	36.2	128/75	53	No	No

Abbreviations: acute coronary syndrome (ACS), atrial fibrillation (AF), arterial hypertension (AH), atrioventricular block (AVB), body temperature (BT), blood pressure (BP), chronic kidney disease (CKD), infusion associated reaction (IAR), hypertrophic cardiomyopathy (HCM), heart rate (HR), ST-elevation myocardial infarction (STEMI), and transient ischaemic attack (TIA). IAR included cutaneous rash, pruritus, anaphylaxis, headache, dyspnoea, urticaria, or any other relevant reaction.

**Table 3:** Physical examination performed and questions asked at each infusion by a medical doctor at the patient’s bedside.

Patient Number	Naïve to ERT (0 = No/1 = Yes)	Physical Examination			Questions			
		Anaphylaxis	Cutaneous Rash	Fever	Pruritus During the 90-min Infusion	Dyspnoea During the 90-min Infusion	Asthenia During the 90-min Infusion	Patient Satisfied About the Reduced Infusion Time
P#1	0	No	No	No	No	No	No	Yes
P#2	0	No	No	No	No	No	No	Yes
P#3	0	No	No	No	No	No	No	Yes
P#4	1	No	No	No	No	No	No	Yes
		No	No	No	No	No	No	Yes
		No	No	No	No	No	No	Yes
		No	No	No	No	No	No	Yes
		No	No	No	No	No	No	Yes
		No	No	No	No	No	No	Yes
P#5	0	No	No	No	No	No	No	Yes
P#6		No	No	No	No	No	No	Yes
		No	No	No	No	No	No	Yes
		No	No	No	No	No	No	Yes
	0	No	No	No	No	No	No	Yes
		No	No	No	No	No	No	Yes
		No	No	No	No	No	No	Yes
		No	No	No	No	No	No	Yes
P#7	0	No	No	No	No	No	No	Yes
		No	No	No	No	No	No	Yes
		No	No	No	No	No	No	Yes
		No	No	No	No	No	No	Yes
		No	No	No	No	No	No	Yes
		No	No	No	No	No	No	Yes
		No	No	No	No	No	No	Yes
		No	No	No	No	No	No	Yes
		No	No	No	No	No	No	Yes
P#8	0	No	No	No	No	No	No	Yes
		No	No	No	No	No	No	Yes
P#9	1	No	No	No	No	No	No	Yes
P#10	1	No	No	No	No	No	No	Yes
		No	No	No	No	No	No	Yes
P#11		No	No	No	No	No	No	Yes
	0	No	No	No	No	No	No	Yes
		No	No	No	No	No	No	Yes
P#12	1	No	No	No	No	No	No	Yes
		No	No	No	No	No	No	Yes
		No	No	No	No	No	No	Yes
		No	No	No	No	No	No	Yes
		No	No	No	No	No	No	Yes
		No	No	No	No	No	No	Yes
P#13	1	No	No	No	No	No	No	Yes
		No	No	No	No	No	No	Yes
		No	No	No	No	No	No	Yes
P#14	0	No	No	No	No	No	No	Yes
P#15	0	No	No	No	No	No	No	Yes
P#16	0	No	No	No	No	No	No	Yes
P#17	0	No	No	No	No	No	No	Yes
		No	No	No	No	No	No	Yes
		No	No	No	No	No	No	Yes
		No	No	No	No	No	No	Yes
P#18	0	No	No	No	No	No	No	Yes
P#19	0	No	No	No	No	No	No	Yes
P#20	0	No	No	No	No	No	No	Yes
		No	No	No	No	No	No	Yes
		No	No	No	No	No	No	Yes
P#21	0	No	No	No	No	No	No	Yes
		No	No	No	No	No	No	Yes
P#22	0	No	No	No	No	No	No	Yes
P#23	0	No	No	No	No	No	No	Yes
P#24	0	No	No	No	No	No	No	Yes
P#25	0	No	No	No	No	No	No	Yes
P#26	0	No	No	No	No	No	No	Yes
P#27	0	No	No	No	No	No	No	Yes
P#28	0	No	No	No	No	No	No	Yes
P#29	0	No	No	No	No	No	No	Yes
P#30	0	No	No	No	No	No	No	Yes
P#31	0	No	No	No	No	No	No	Yes
P#32	0	No	No	No	No	No	No	Yes
P#33	1	No	No	No	No	No	No	Yes
		No	No	No	No	No	No	Yes
		No	No	No	No	No	No	Yes
P#34	0	No	No	No	No	No	No	Yes
P#35	0	No	No	No	No	No	No	Yes
P#36	0	No	No	No	No	No	No	Yes
P#37	0	No	No	No	No	No	No	Yes
P#38	0	No	No	No	No	No	No	Yes
P#39	0	No	No	No	No	No	No	Yes

## Data Availability

All data underlying this publication are available in the article.
